# Adaptive Image Rendering Using a Nonlinear Mapping-Function-Based Retinex Model

**DOI:** 10.3390/s19040969

**Published:** 2019-02-25

**Authors:** JongGeun Oh, Min-Cheol Hong

**Affiliations:** School of Electronic Engineering, Soongsil University, Seoul 06978, Korea; confisio@ssu.ac.kr

**Keywords:** image rendering, nonlinear mapping function, retinex model, statistics, contrast ratio

## Abstract

This paper introduces an adaptive image rendering using a parametric nonlinear mapping-function-based on the retinex model in a low-light source. For this study, only a luminance channel was used to estimate the reflectance component of an observed low-light image, therefore halo artifacts coming from the use of the multiple center/surround Gaussian filters were reduced. A new nonlinear mapping function that incorporates the statistics of the luminance and the estimated reflectance in the reconstruction process is proposed. In addition, a new method to determine the gain and offset of the mapping function is addressed to adaptively control the contrast ratio. Finally, the relationship between the estimated luminance and the reconstructed luminance is used to reconstruct the chrominance channels. The experimental results demonstrate that the proposed method leads to the promised subjective and objective improvements over state-of-the-art, scale-based retinex methods.

## 1. Introduction

The high performance and miniaturization of image sensors make it possible for image information to be used in various applications, such as mobile platforms, recognition systems, and security systems [1,2]. However, low contrast coming from an absent light source leads to the degradation of image quality, so the performance of the application system may be unsatisfactory [3]. In order to solve the low-contrast problem, many simple approaches, such as histogram equalization, gamma correction, and auto exposure, have been widely used [4]. However, they limit performance because they do not account for human visual perception [5].

Many efforts have been made to formalize human visual systems (HVSs). Among them, retinex theory has attracted attention as a useful way to estimate the human sensation derived from an observed scene. For example, Land et al. presented a model of HVS color perception. It explains how an HVS, as a combination of processes, supposedly taking place in both the retina and the cortex, is capable of adaptively coping with illumination that varies spatially in both intensity and color [6].

Enhancements of low-contrast images using the retinex model are aimed at estimating illuminance and reflectance under various assumptions. According to the mathematical formulation and implementation-of-cost function, these can be classified as modified retinex methods [7,8,9], scale-based methods [10,11,12,13,14,15], variational methods [16,17,18], and deep learning-based methods [19,20]. The modified retinex methods use a reset and threshold mechanism to estimate illuminance based on the pixel intensity of a given random path. These methods are robust against additive noise. However, they are limited in improving the contrast ratio because they do not account for the statistical distribution of low-light images. The variational methods, which model appropriate energy functions, have led to promising results. However, their performance is very sensitive to tuning function. In addition, they require very expensive computational costs, so the scope of their applications is limited. Recently, the deep learning-based methods have been exploited to enhance the contrast ratio. Most of the schemes are based on the property of the linear retinex model. Therefore, in order to improve the performance of the deep learning approaches, based on the retinex model, it is necessary to study the retinex model that reflects HVS. 

A single-scale retinex (SSR) method has been introduced, in which a center/surround Gaussian filter is used to extract the reflectance from an observed image in accordance with the Werber-Fachner Law and based on the nonlinearity of human visual perception. This leads to an enhancement of the contrast range [11]. However, the performance is very sensitive to the choice of parameters for the Gaussian filter. A multi-scale retinex (MSR) model and an MSR with color restoration (MSRCR) model have been presented to resolve the filter dependency problem [12]. They have the capability to effectively enhance contrast ratios with less filter dependency, but they also increase the number of halo artifacts, which is visually annoying. The artifacts increase, as the number of filters increase.

An adaptive MSR (AMSR) [21] was created to improve the contrast ratio and reduce the color distortion; in this system, luminance is used to estimate the reflectance from an observed image. The estimated reflectance is used to reconstruct reflectance via linear stretching assisted by a weighted map. Although the AMSR improves the contrast ratio and reduces computational complexity, it increases the number of halo artifacts because the statistical properties of the extracted reflectance are not incorporated into the reconstruction process.

The bottlenecks of the existing scale-based retinex methods are summarized as follows: (1) the number of halo artifacts due to the use of multiple center/surround Gaussian filters, (2) color distortion due to independent processing of color channels, and (3) loss of signal distribution characteristics due to not considering the statistics of the observed images.

**Figure 1:**
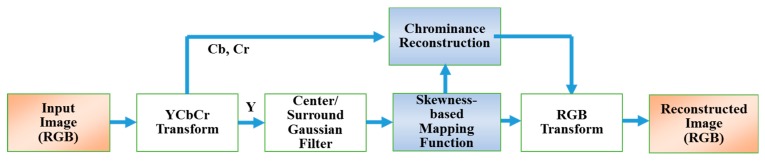
Flowchart of the proposed method.

This paper presents an image rendering method via an adaptive, scale-based retinex model using a parametric, nonlinear mapping function of statistical characteristics of luminance and reflectance for low-light images. In order to reduce the number of halo artifacts, a center/surround Gaussian filter is only applied to the luminance channel in the YCbCr color space to estimate the reflectance. The statistical characteristics of the captured image are distributed differently according to the brightness and direction of the light source. Therefore, it is necessary to incorporate these statistical characteristics into the reconstruction process of the reflectance. This paper introduces a nonlinear reflectance reconstruction function that is defined as a function of the skewness of the luminance of a low-light image, so the contrast ratio is adaptively controlled. In addition, a new determination of the gain and offset of the nonlinear function is addressed to adaptively clip the dynamic range of the reflectance. Finally, the chrominance channels are reconstructed by the ratio between the estimated luminance and the reconstructed luminance. Figure 1 depicts the overall flowchart of the proposed method.

This paper is organized as follows. Section 2 briefly describes the MSR for low-light contrast enhancement. Section 3 describes the proposed scale-based retinex method using a new parametric, nonlinear function for enhancing low-light images. The determination of parameters, the gain, and the offset of the nonlinear function using the statistical characteristics are explained in this section as well. We analyze the experimental results in Section 4, and finally, describe the conclusions derived from the results in Section 5.

## 2. Related Work

The human visual model has been well studied in regard to solving low-light and back-light problems. Land, et al. experimentally proved that the human visual model can be expressed by the reflection coming through an object and the illuminance coming from a light source [6]. According to their research, perceptual intensity can be expressed as

(1) I=R·L,

where *I*, *R*, and *L* represent the perceptual intensity of human eyes, the reflectance, and the illuminance, respectively. Equation (1) implies that the illuminance and the reflectance can be arithmetically obtained. Based on the retinex theory, many approaches have been presented to obtain better results by reconstructing the reflectance or the illuminance. The SSR method aimed to correct the reflectance of an object by applying center/surround Gaussian filters to an observed image as follows [11]:

(2) R(x,y)=logI(x,y)−log(I(x,y)∗G(x,y)),

where ∗ denotes the two-dimensional convolution operator, and *G* represents a Gaussian filter. The Gaussian filter of the (x,y)-th pixel is defined as follows:

(3) $$G\left(x,y\right)=Ke^{-\left({\left(x-x^{\prime }\right)}^{2}+{\left(y-y^{\prime }\right)}^{2}\right)/c^{2}},\left(x^{\prime },y^{\prime }\right)\in S,$$

where *K* and *c* denote a normalization constant and standard deviation, respectively, and *S* represents a two-dimensional support region to which the Gaussian filter is applied. The above expression means that the density of light concentrates around the light source, and the correlation of light decreases as the distance from the center increases. It was verified that the SSR method is very sensitive to the choice of standard deviation *c* [22].

In order to solve this problem, an MSR method was proposed in which *N* center/surround Gaussian filters are applied to each channel of an input color image and weights are applied to each result to reduce the dependency of the filter. The reflection of the *i*-th color channel is estimated as follows [12]:

(4) $$R_{i}^{MSR}\left(x,y\right)=\overset{N}{\underset{n=1}{\sum }}w_{n}log\left[\frac{I_{i}\left(x,y\right)}{I_{i}\left(x,y\right)*G\left(x,y\right)}\right]$$

for i∈{R,G,B}. In equation (4), N=3 is generally used because the computational cost increases as N increases. It has been shown that $w_{n}$ = {0.3, 0.1, 0.6} and $c_{n}=\left\{5,30,240\right\}$ are effective for obtaining a reasonable result [23]. The estimated reflectance, $R_{i}^{MSR}$, includes distorted color and illuminance components, so a gain/offset is set to reconstruct the reflection as follows:

(5) $${\hat{R}}_{i}^{MSR}\left(x,y\right)=\max\left[\min\left\{\left(\frac{R_{i}^{MSR}\left(x,y\right)-R_{i,\;min}^{MSR}}{R_{i,\;max\;}^{MSR}-R_{i,\;min}^{MSR}}\right),1\right\},0\right],$$

where $R_{i,max}^{MSR}$ and $R_{i,min}^{MSR}$ represent the maximum and the minimum, respectively, of the estimated reflectance and are determined using statistical characteristics as follows:

(6) $$R_{i,max}^{MSR}=max\left(m_{i}+\alpha \sigma_{i},1\right),\;R_{i,min}^{MSR}=min\left(m_{i}-\alpha \sigma_{i},0\right),$$

where α represents a constant to clip the dynamic range. In addition, $m_{i}$ and $\sigma_{i}$ denote the mean and standard deviation of $R_{i}^{MSR}$, respectively. For an image represented by *k* bits per pixel, each pixel is reconstructed as follows:

(7) $${\hat{I}}_{i}\left(x,y\right)={\hat{R}}_{i}^{MSR}\left(x,y\right)\times \left(2^{k}-1\right).$$

It has been shown that MSR methods have the capability of reducing filter dependency, but they also increase the number of halo artifacts caused by the independent processing of the center/surround Gaussian filters to RGB channels. In addition, there is a limit to improvements to the contrast ratio because the statistical characteristics of the energy density of the observed low-light image are not considered.

## 3. The Proposed Method

In order to solve the problems of the existing scale-based retinex methods, this paper presents an adaptive scale-based retinex model based on a nonlinear function using the skewness characteristics of luminance and reflectance. The luminance channel (Y) in the YCbCr color space is suitable to represent the perceptual information and to include the relationships between RGB channels. Therefore, reflectance can be estimated by applying the center/surround Gaussian filter to only the luminance. Therefore, the number of halo artifacts and computational complexity can both be reduced. Skewness has been used to statistically represent the degree of bias of energy density. In this paper, a nonlinear function, defined as a function of the mean, variance, and skewness of the estimated reflectance and luminance, is presented to improve the contrast ratio and reduce the number of halo artifacts.

In the study, an observed low-light RGB image is transformed to the YCbCr image, and the reflectance of Y channel is obtained in a similar way to MSR methods as follows:

(8) $$R\left(x,y\right)=\sum_{n=1}^{N}w_{n}log\left[\frac{Y\left(x,y\right)}{Y\left(x,y\right)*G\left(x,y\right)}\right],$$

where Y and G denote the luminance channel and the center/surround Gaussian filter, respectively. In addition, N=3 is used with $w_{n}$ = {0.3, 0.1, 0.6} and $c_{n}=\left\{5,30,240\right\}$ in the same way as the MSR. 

As mentioned, the conventional scale-based retinex methods have limited performance because they do not incorporate the statistical characteristics of the energy density of an observed image into the reconstruction process. In this study, skewness is used to represent the bias degree of the energy density. For a U×V-sized image, the skewness of the luminance and the estimated reflectance can be written as follows [24]:

(9) $$\begin{array}{c} Sk_{Y}=\frac{1}{UV}\sum_{x=0}^{U-1}\sum_{y=0}^{V-1}{\left[\frac{Y\left(x,y\right)-m_{Y}}{\sigma_{Y}}\right]}^{3},\\ Sk_{R}=\frac{1}{UV}\sum_{x=0}^{U-1}\sum_{y=0}^{V-1}{\left[\frac{R\left(x,y\right)-m_{R}}{\sigma_{R}}\right]}^{3}, \end{array}.$$

where $m_{Y}$ and $\sigma_{Y}$ denote the mean and the standard deviation, respectively, of the luminance, and $m_{R}$ and $\sigma_{R}$ represent the mean and standard deviation of the estimated reflectance, respectively.

**Figure 2:**
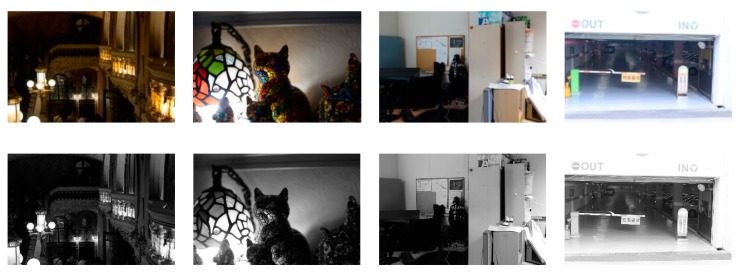

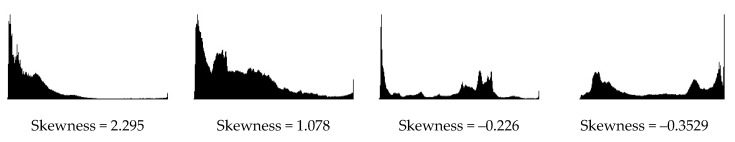
Examples of histogram and skewness: (from top to bottom) low-light color image, luminance image, and histogram and skewness of luminance image.

As shown in Figure 2, the skewness increases as the luminance becomes darker. In addition, the skewness is equal to 0 when the distribution is symmetrical. As the amount of the available light-source lessens, there is a distortion of the illuminance and the estimated reflectance [11,12]. Therefore, it is necessary to compensate for the distortion. In conventional approaches, the linear compensation in equation (5) is used, but there is a limit to the ability to correct the distortion with this equation because the statistical characteristics of the observed image are not reflected. Therefore, a new reconstruction function is used as follows:

(10) $$\hat{R}\left(x,y\right)=\max\left[\min\left\{{\left(\frac{R\left(x,y\right)-R_{min}}{R_{max}-R_{min}}\right)}^{\mu },1\right\},0\right],$$

where $R_{max}$ and $R_{min}$ are the maximum and minimum for the gain and offset of the estimated reflectance, respectively. In order to improve the contrast ratio, reflectance should be expanded by setting μ to be larger, as the image becomes darker. Conversely, μ is set to decrease, as the image gets brighter so the reflectance becomes compressed. The relationship between μ and $Sk_{Y}$ can be written as follows:

(11) $$\mu \propto \{\begin{array}{c} Sk_{Y}\mathrm{for}Sk_{Y}\geq 0,\\ \frac{1}{\left|Sk_{Y}\right|}\mathrm{for}Sk_{Y}<0, \end{array}$$

where satisfying equation (11) with μ can be justified in various ways. In this study, μ is defined as a function of $Sk_{Y}$ as follows:

(12) $$\mu =\{\begin{array}{cc} 1+\left(\alpha \;\times Sk_{Y}\right) & \mathrm{if}Sk_{Y}\geq 0,\\ \frac{1}{\left(1+\;\alpha \;\times \left|Sk_{Y}\right|\right)} & \mathrm{otherwise}, \end{array}$$

where α is a constant.

In the MSR, the gain and offset in equation (6) are determined only by the mean and standard deviation of the estimated reflection, under the assumption that the estimated reflection has a bilateral symmetrical distribution. However, the distribution of the estimated reflection is not symmetrical because the estimated reflectance may contain a distorted component that is dependent on light intensity. Therefore, it is necessary to set the gain and offset by the degree of asymmetry of the reflectance. In this study, they are defined as follows:

(13) $$\begin{array}{c} R_{max}=m_{R}+\sigma_{R}\times \left(T+\beta \times Sk_{R}\right),\\ R_{min}=m_{R}-\sigma_{R}\times \left(T+\beta \times Sk_{R}\right), \end{array}$$

where β is a constant to scale the skewness. In addition, the constant, T, is chosen such that $\left(T+\beta \times Sk_{R}\right)$ is greater than 0. Equations (10) and (13) have the following properties. When the skewness of the estimated reflection is positive, the estimated reflection is concentrated in a lower-than-average reflectance region. In this case, $R_{max}$ and $R_{min}$ are determined to expand the concentrated reflectance region. Conversely, $R_{max}$ and $R_{min}$ are chosen to expand a higher-than-average reflectance region when the skewness is negative. According to these properties, the dense and loose regions of the estimated reflectance are reconstructed in a balanced manner. Then, the luminance of a pixel represented by k bits is reconstructed in the following manner:

(14) $$\hat{Y}\left(x,y\right)=\hat{R}\left(x,y\right)\times \left(2^{k}-1\right).$$

The chrominance channels corresponding to the reconstructed luminance can be reconstructed in various ways. In this study, the chrominance channels are reconstructed by gains in luminance in order to maintain the correlation between the channels with the reduction of the computational cost. The gains in luminance can be defined as follows:

(15) $$\rho \left(x,y\right)=\frac{\hat{Y}\left(x,y\right)}{Y\left(x,y\right)}\times \gamma ,$$

where γ is a constant. Then, Cb and Cr are reconstructed as follows:

(16) $$\hat{Cb}\left(x,y\right)=\rho \left(x,y\right)\times \left(Cb\left(x,y\right)-128\right)+128,\;\hat{Cr}\left(x,y\right)=\rho \left(x,y\right)\times \left(Cr\left(x,y\right)-128\right)+128.$$

## 4. Experimental Results

### 4.1. Experimental Setup

**Figure 3:**
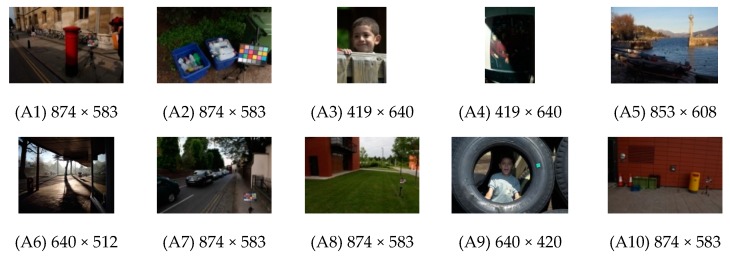

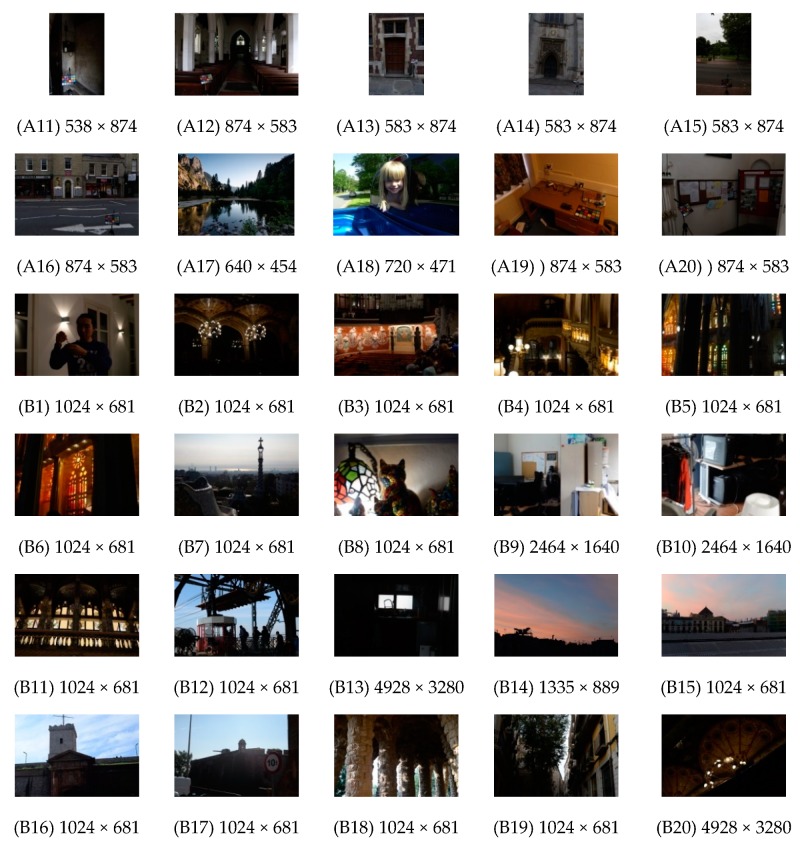
Test images used in the experiments.

Several experiments were conducted with various low-contrast images, such as indoor/outdoor environments and single/multiple light sources. As shown in Figure 3, for the experiments, 20 images (A1‒A20) were obtained from the Internet and 20 images (B1-B20) were acquired with a Nikon-Df camera using AF-S NIKKOR 50 mm f/1.8 G lens.

The proposed method was compared to the state-of-the-art, scale-based retinex algorithms, such as the MSR [12], random spray retinex (RSR) [7], light RSR (LRSR) [8], and AMSR [21]. To evaluate the performance of the algorithms, contrast per pixel (CPP) [25] was used. For a U×V-sized color image, the CPP is defined as follows:

(17) $$\mathrm{CPP}=\frac{\sum_{k=1}^{3}\sum_{i=0}^{U-1}\sum_{j=0}^{V-1}\left(\frac{1}{9}\sum_{m=-1}^{1}\sum_{n=-1}^{1}\left|{\hat{I}}_{k}\left(i,j\right)-{\hat{I}}_{k}\left(i+m,j+n\right)\right|\right)}{U\times V}$$

where ${\hat{I}}_{k}$ (k=1,2,3) represents the k-th reconstructed channel of an RGB color image. An Intel Core i7-3770 CPU 3.4GHz with 8 GB memory was used to examine the processing time, and MS C++ 2010 was used to simulate the algorithms. To evaluate subjective visual quality, a double-stimulus continuous quality scale (DSCQS) [26] was examined, with which a blind quality assessment was conducted by 20 individuals.

Several parameters were defined for the proposed method. α and β in equations (12) and (13) were used to reflect the contribution of the skewness of the luminance and the reflectance in the mapping function. As they increase, the contrast ratio of the reconstructed image showed an out-of-proportion increase as well. It was observed that 1.5≤α,β≤2.5 led to promising results, and α=β=2 was used to reconstruct the image. Additionally, T in equation (13) was used to set the gain and offset of the mapping function. As T decreased, the degree of the saturation of the brightness increased. Conversely, as T increased, the contrast ratio of the reconstructed image decreased, so the saturation and the brightness were both reduced. In these experiments, T=2 was used. In addition, the luminance gain, γ in equation (15), was used to reconstruct the Cb and Cr channels. As γ increased, the chrominance channels became more saturated. The experiments yielded 0.85<γ<0.95, which is a good range with respect to performance. In these experiments, γ=0.9 was used.

### 4.2. Analyses of Experimental Results

The CPP has been used previously as a way to represent the degree of intensity variation between neighbor pixels, and it has been shown to decrease as the contrast ratio of an observed image decreases [23]. Table 1 shows the CPP comparisons for this study. With the conventional MSR method, improvements in CPP varied depending on the image. Conversely, the RSR and LRSR methods were very effective for noise reduction in the low-contrast region, but they were limited in improving CPP. AMSR outperformed the other methods in terms of the CPP in most cases. However, it was observed that the CPP improvement was caused by a halo artifact increase. Conversely, the proposed method outperformed the comparative methods, with the exception of AMSR. It was observed that the proposed method led to better, consistently guaranteed results with respect to CPP, regardless of the degree of contrast. In these experiments, the average CPP improvements for the low-light images, MSR, AMSR, RSR, LRSR, and the proposed method were 78.9%, 134.2%, 7.7%, 7.7%, and 113.1%, respectively.

The comparisons of the processing times per pixel are presented in Table 2. In AMSR and the proposed method, the processing times for converting the RGB ground truth image into the YCbCr channels and converting the reconstructed YCbCr channels into the RGB image are included. The MSR required more computation than the proposed method due to the independent reconstruction processing for each channel. Additionally, the computational complexities of the RSR and LRSR were the most expensive due to the large number of random spray filters, and the filter window size applied to each pixel. The AMSR required less computation than the other comparative methods because it performed the Y-channel oriented processing. However, it spent a certain amount of processing time to reconstruct the chrominance channels, revealing marginally higher computational complexity than the proposed method. Conversely, it was confirmed that the proposed method consistently had the lowest computational cost of all the methods because it directly applied the statistical characteristics of an observed image to the mapping function. The processing time reductions of the proposed method over the MSR, AMSR, RSR, and LRSR were 100.4%, 14.3%, 249.3%, and 263.8%, respectively.

**Figure 4:**
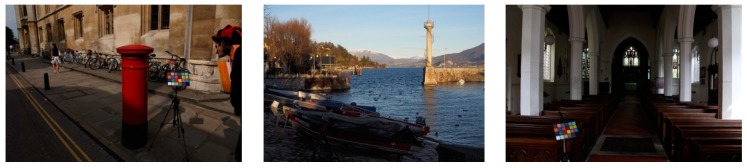

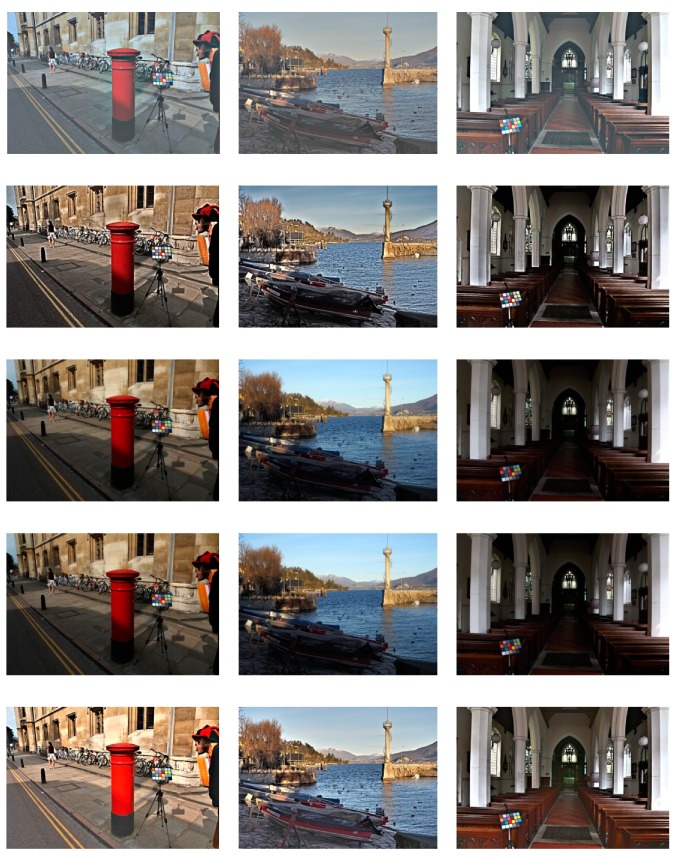
Visual comparisons with A1, A5 and A12 test images: (from top to bottom) low-light image, MSR, AMSR, RSR, LRSR, and proposed method.

**Figure 5:**
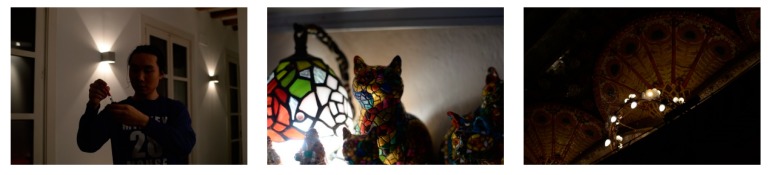

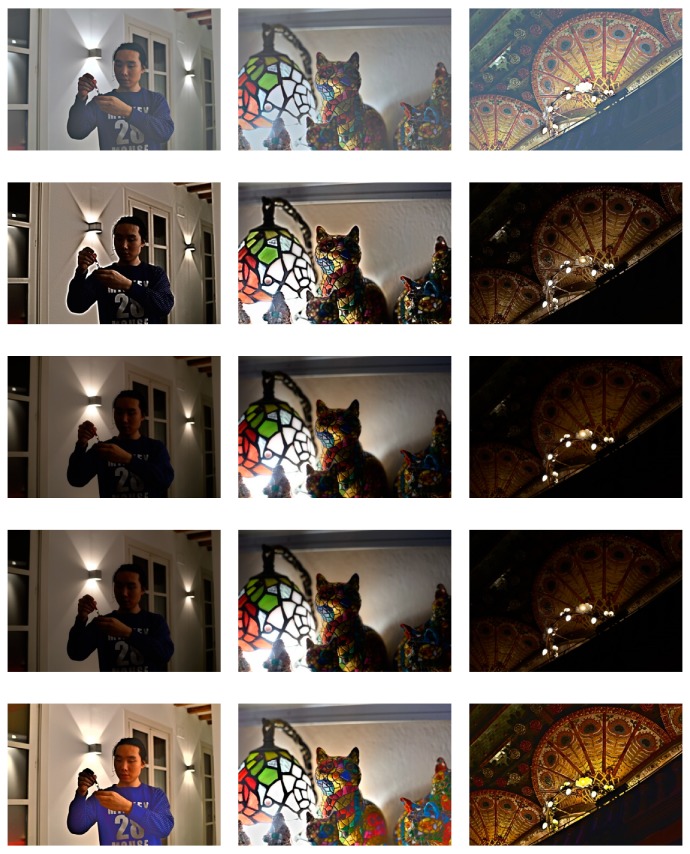
Visual comparisons with B1, B8, and B20 test images: (from top to bottom) low-light image, MSR, AMSR, RSR, LRSR, and proposed method.

Visual comparisons are presented in Figure 4 and Figure 5. The MSR was effective in improving the contrast ratio. However, there was signal saturation and color distortion because this method did not consider the statistical characteristics of the observed image in reconstructing the reflectance. Although the AMSR was better than the MSR in terms of the contrast ratio, the number of halo artifacts increased because the linear stretching assisted by a weighted map, without considering the asymmetry of the reflectance of the observed image. The RSR and LRSR were effective in color representation and they removed noise in low-contrast regions well. However, they were limited in their ability to enhance the contrast ratio. Conversely, the proposed method considered the distribution characteristics of the image, thereby improving the contrast ratio and effectively representing the color components.

Table 3 illustrates the comparisons of the DSCQS for subjective quality assessment, in which the low-light image was assumed to be 5 points and 0-10 points were used to score the compared image. In most cases, the MSR scores were higher than the comparative methods, but there was a large difference in the evaluators’ preferences, depending on the images. AMSR had the lowest score among the comparative methods due to the number of halo artifacts, although it outperformed the others in terms of CPP. These experiments verified that the number of halo artifacts was an important cause of visual inconvenience. RSR and LRSR had relatively low scores due to the performance limits in improving the contrast ratio. On the other hand, the proposed method adaptively improved the contrast ratio with the reduction of the color distortion, leading to it consistently outperforming the other methods.

The experiments proved that, subjectively and objectively, promising results were obtained by incorporating the asymmetry of the extracted reflectance and the illuminance into the reconstruction process. The experiments confirmed that the objective performance evaluation, CPP, did not coincide with the subjective performance evaluation, such as the DSCQS, because CPP does not consider the halo artifacts and the color distortion. Therefore, it is necessary to study a quality assessment metric that reflects the elimination of the halo artifact and the improvement of color distortion, as well as the improvement of the contrast ratio. 

## 5. Conclusions

This paper presents an adaptive image rendering method using the asymmetry of an observed image in a low-light environment. A new nonlinear mapping function, as determined by the asymmetry of the illuminance, and the extracted reflectance, was presented for reconstructing the reflectance. In addition, the determination of the gain and offset of the nonlinear mapping function was also introduced. The experimental results demonstrated that the proposed method leads to subjectively and objectively promising results. The proposed method can be used as a computational platform to provide the high-quality image in various vision-sensor-based intelligent systems, such as visual surveillance and vision assistant driving systems, in a low-light source environment. 

In these experiments, halo artifacts were the main cause of increased CPP, but at the same time, the artifacts were very annoying to human viewers. Therefore, it is worth developing an objective image quality assessment to consider the elimination of the halo artifact and the color distortion, as well as the improvement of the contrast ratio. A new, high-order, norm-based, deep learning method assisted by asymmetrical characteristics is under development, and the newest method is expected to produce a more sophisticated formulation and achieve even better performance.

## Figures and Tables

**Table 1:** CPP comparisons.

Image	Low-Light Image	MSR [12]	AMSR	RSR [7]	LRSR [8]	Proposed Method
A1	49.15	107.10	**125.08**	57.14	57.91	108.80
A2	93.48	123.61	**161.08**	61.18	61.44	135.53
A3	64.40	80.68	**106.13**	51.77	51.75	87.24
A4	39.58	43.28	47.31	25.19	25.20	**52.65**
A5	64.89	145.21	**195.21**	86.61	86.13	156.65
A6	74.37	104.75	**130.80**	75.50	75.50	121.39
A7	81.59	139.97	**168.05**	90.83	90.81	147.96
A8	47.61	131.13	**174.43**	66.30	66.50	132.10
A9	50.94	86.63	**122.18**	47.76	47.72	91.02
A10	23.59	127.66	**140.02**	60.76	59.63	139.79
A11	20.25	53.63	**63.24**	22.01	22.06	59.34
A12	40.53	71.90	**92.44**	40.59	40.55	79.46
A13	46.69	114.50	**150.67**	66.90	66.52	116.76
A14	50.07	156.64	**171.69**	70.32	70.50	158.31
A15	44.55	86.26	**109.51**	48.20	48.16	88.11
A16	74.68	174.04	**201.31**	80.90	80.90	182.31
A17	102.04	150.07	**198.70**	102.34	102.32	162.68
A18	58.23	91.08	**121.71**	58.23	58.24	101.36
A19	26.10	72.53	**92.45**	35.39	35.39	72.83
A20	22.39	72.34	**88.95**	51.13	51.41	77.18
B1	18.10	34.02	**62.89**	18.10	18.10	54.72
B2	37.21	64.25	85.68	37.21	37.21	**171.48**
B3	72.29	92.70	157.57	72.33	72.38	**159.09**
B4	43.39	57.35	**102.68**	43.39	43.39	101.91
B5	42.18	53.55	78.27	42.18	42.18	**93.28**
B6	30.44	45.33	62.39	30.47	30.49	**84.21**
B7	32.34	37.73	**74.18**	32.94	32.94	64.44
B8	29.93	34.14	**61.00**	29.93	29.93	55.09
B9	17.82	22.19	**51.64**	17.91	17.91	34.55
B10	23.73	26.08	**53.05**	23.73	23.73	46.56
B11	57.31	85.71	**103.21**	57.51	57.40	92.46
B12	70.76	85.56	**107.85**	75.56	75.46	102.88
B13	7.16	26.70	18.09	7.23	7.23	**28.90**
B14	7.29	15.96	**19.41**	11.15	11.16	15.58
B15	38.22	83.06	**132.15**	46.55	46.58	87.75
B16	31.78	54.21	**80.94**	32.77	32.78	63.38
B17	29.58	47.03	**58.78**	31.14	31.05	50.82
B18	89.18	132.21	**163.30**	89.18	89.18	147.28
B19	93.72	144.15	165.91	93.72	93.74	**172.37**
B20	10.54	48.49	38.88	10.54	10.54	**54.72**
**Average**	45.80	81.97	**107.28**	49.33	49.31	97.58

**Table 2:** Processing-time per pixel comparisons (unit: microsecond).

Image	MSR	AMSR	RSR	LRSR	Proposed Method
A1	6.231	3.584	11.255	11.771	**3.109**
A2	6.257	3.560	11.155	11.673	**3.097**
A3	6.254	3.520	11.210	11.687	**3.043**
A4	6.179	3.502	11.232	11.691	**3.050**
A5	7.641	4.076	12.738	13.252	**3.571**
A6	7.108	4.352	12.756	13.208	**3.650**
A7	6.241	3.588	11.206	11.642	**3.099**
A8	7.583	4.172	12.688	13.316	**3.586**
A9	6.135	3.601	11.094	11.581	**3.147**
A10	6.306	3.841	11.122	11.658	**3.709**
A11	7.114	3.213	10.767	11.098	**2.751**
A12	5.962	3.185	10.704	11.141	**2.761**
A13	5.895	3.175	10.578	11.230	**2.907**
A14	5.992	3.299	10.910	11.453	**2.856**
A15	6.245	3.195	10.716	11.283	**2.871**
A16	5.929	3.246	10.922	11.102	**2.765**
A17	5.909	3.380	10.611	10.845	**2.870**
A18	5.653	3.111	10.468	11.011	**2.728**
A19	5.952	3.158	10.513	10.982	**2.748**
A20	5.952	3.132	10.472	10.982	**2.742**
B1	6.999	3.813	10.976	11.311	**3.311**
B2	5.978	3.718	10.888	11.347	**3.489**
B3	6.120	3.710	10.870	11.375	**3.262**
B4	6.017	3.835	10.791	11.293	**3.221**
B5	5.955	3.781	10.904	11.332	**3.252**
B6	6.007	3.747	10.838	11.277	**3.267**
B7	6.052	3.681	10.794	11.322	**3.292**
B8	6.019	3.694	10.890	11.290	**3.196**
B9	6.917	3.825	10.885	11.390	**3.363**
B10	6.916	3.783	10.913	11.376	**3.181**
B11	6.041	3.677	10.754	11.330	**3.138**
B12	6.003	3.598	10.553	11.075	**3.251**
B13	8.147	3.791	11.963	12.308	**3.462**
B14	5.954	3.373	10.608	11.015	**2.989**
B15	5.910	3.588	10.636	11.019	**3.129**
B16	6.062	3.612	10.584	10.995	**3.254**
B17	5.935	3.923	10.629	10.970	**3.099**
B18	5.894	3.523	10.583	10.980	**3.126**
B19	5.894	3.597	10.537	10.953	**3.252**
B20	6.984	3.850	11.277	11.679	**3.378**
**Average**	6.309	3.600	11.000	11.456	**3.149**

**Table 3:** DSCQS comparisons.

Image	MSR		AMSR		RSR		LRSR		Proposed	
	Avg.	Std.	Avg.	Std.	Avg.	Std.	Avg.	Std.	Avg.	Std.
A1	5.556	2.672	4.556	2.076	5.444	1.824	5.611	1.857	**6.833**	2.256
A2	5.444	2.289	4.556	1.774	5.944	1.359	6.278	1.236	**5.667**	2.220
A3	4.778	2.269	5.167	2.269	5.111	1.431	5.111	1.023	**7.167**	1.833
A4	5.000	2.238	3.444	1.749	5.611	1.241	5.722	1.229	**6.556**	1.562
A5	5.889	1.609	3.889	1.813	4.500	1.200	4.667	1.071	**6.333**	2.012
A6	**7.444**	1.744	4.444	1.946	5.500	1.161	5.611	1.152	7.389	1.396
A7	4.944	2.159	4.778	2.128	5.111	0.805	5.167	0.768	**5.556**	2.308
A8	6.667	1.952	3.278	1.905	5.722	1.382	5.667	1.236	**6.668**	1.749
A9	4.056	1.359	4.111	1.830	5.611	1.241	5.833	1.400	**6.556**	1.851
A10	6.556	2.617	3.772	1.744	6.056	1.265	6.111	1.284	**6.778**	2.632
A11	4.550	2.523	3.350	2.207	5.650	1.565	5.850	1.531	**6.250**	2.268
A12	5.100	2.245	4.650	2.207	5.050	1.432	4.550	1.191	**7.450**	1.638
A13	6.900	1.744	4.300	1.780	6.800	1.240	5.800	1.673	**8.250**	1.446
A14	7.200	1.473	3.850	1.309	6.450	1.572	4.600	1.875	**7.250**	2.221
A15	5.850	2.033	4.450	1.872	5.350	1.089	4.750	1.293	**6.500**	2.115
A16	**7.200**	1.795	4.300	1.559	5.350	0.998	5.100	1.210	7.000	1.864
A17	5.300	2.003	4.500	1.821	5.600	1.392	5.050	1.146	**6.150**	2.300
A18	5.700	1.809	4.200	1.542	5.150	1.424	5.000	1.487	**5.950**	2.502
A19	6.800	1.436	4.000	1.589	5.950	1.432	5.850	1.599	**7.500**	2.103
A20	6.400	1.501	3.700	1.525	7.600	1.392	7.000	1.451	**8.250**	1.585
B1	7.113	0.816	3.111	0.994	4.444	1.066	4.333	0.943	**7.333**	0.943
B2	5.556	2.061	5.111	0.875	4.556	0.685	4.667	0.667	**7.111**	1.286
B3	7.111	1.728	5.889	1.286	5.333	0.471	5.222	0.629	**7.667**	0.943
B4	5.667	2.000	5.556	1.423	5.111	0.314	5.111	0.314	**6.556**	1.707
B5	6.778	1.750	5.889	1.100	4.889	0.737	4.989	0.750	**7.778**	1.685
B6	3.667	1.633	5.556	0.956	4.778	0.629	4.987	0.692	**7.111**	0.567
B7	**7.556**	1.257	3.889	1.523	5.222	0.786	5.111	0.567	7.000	0.943
B8	7.333	1.826	5.444	1.257	4.889	0.567	4.889	0.567	**7.444**	0.685
B9	7.556	1.872	3.889	1.286	5.000	0.667	4.889	0.567	**7.778**	1.771
B10	7.000	0.943	4.889	1.100	4.778	1.100	4.889	0.567	**7.111**	1.523
B11	4.350	1.981	5.600	2.062	5.350	1.424	5.050	1.050	**6.200**	2.215
B12	4.300	1.809	4.100	1.651	5.050	0.887	4.800	1.105	**5.050**	2.434
B13	4.800	2.546	4.750	1.333	5.000	0.918	4.600	0.940	**5.200**	2.419
B14	**5.950**	1.986	2.850	1.663	5.600	2.393	4.850	2.110	5.250	2.468
B15	5.900	2.222	3.150	1.531	6.050	1.761	5.750	1.482	**6.600**	2.113
B16	5.250	2.268	3.250	1.517	**5.900**	1.586	5.500	1.318	5.100	2.553
B17	4.450	2.188	3.250	1.618	5.350	1.268	4.900	1.410	**5.900**	2.382
B18	5.400	2.010	5.300	2.452	5.150	1.040	5.000	1.206	**5.600**	2.415
B19	5.150	2.110	5.300	1.525	5.050	1.050	4.800	0.951	**5.350**	2.397
B20	4.750	2.149	5.200	2.215	5.150	0.875	5.000	0.649	**6.150**	2.412
**Average**	5.824	1.916	4.382	1.650	5.405	1.116	5.217	1.125	**6.634**	1.891
